# Additional Porosity as a Side Effect of Polycarboxylate Addition and Its Influence on Concrete’s Scaling Resistance

**DOI:** 10.3390/ma13020316

**Published:** 2020-01-09

**Authors:** Aneta Nowak-Michta

**Affiliations:** Faculty of Civil Engineering, Cracow University of Technology, 24 Warszawska St., 31-155 Cracow, Poland; a_nowak@pk.edu.pl; Tel.: +48-12-628-23-63

**Keywords:** superplasticizer, modified polycarboxylates, air-entrained concrete, air void system, freeze–thaw resistance, scaling, consistency

## Abstract

A side effect of using modified polycarboxylates to liquefy a concrete mix is additional pores in the concrete. They change the air void system in hardened concretes, and can be used to evaluate the freeze–thaw resistance of concretes. The purpose of this study is to determine the impact of the abovementioned quantitative and qualitative parameters on the freeze–thaw resistance of concretes. The research program was performed on eight sets of air-entraining and non-air-entraining concretes with a variable content of superplasticizer based on modified polycarboxylates. The basic composition of and air-entraining admixture content in the air-entraining concrete mixtures were held constant. Pore structure tests were performed according to EN 480-11. Scaling resistance was determined according to PKN-CEN/TS 12390-9. The results showed that as the content of modified polycarboxylates increased, the pore structure was adversely affected, and, consequently, the air void parameters deteriorated. At the same time, the freeze–thaw resistance of the non-air-entraining concretes decreased. The pores sizes also changed. As the fluidity increased, the specific surface area decreased, and, consequently, the spacing factor increased. The air-entraining concretes, despite the deterioration in the pore structure due to the modified polycarboxylates, were found to be very good quality concretes after 56 freeze–thaw cycles in the presence of 3% NaCl.

## 1. Introduction

Ensuring that concrete is resistant to frost is one of the most important durability issues with concrete, and has resulted in numerous research works in this field [1,2,3,4,5,6,7]. Frost–thaw cycles exert two types of destructive effects on concrete. The first is the volumetric effect of frost when de-icing agents are not used. The second type, which is more severe, is the surface effect of frost when defrosting agents are used. This so-called ‘scaling’ is found in road and bridge concretes [1,2,3,4,5,6,7].

The use of thawing salts speeds up the destruction of concrete due to a freeze–thaw cycle. This destruction is most often manifested by chipping on the concrete’s surface. Even when the structure is destroyed due to the very strong effects of a freeze–thaw cycle when thawing agents are used, such destruction only covers the hardened cement paste’s surface layer, which has a thickness of several millimeters [1,6,8,9,10]. The mechanism that underlies the destruction of concrete as a result of peeling is different from the volumetric effect of a freeze–thaw cycle, which occurs as a result of internal crystallization and causes a decrease in strength [4,11].

The durability of the concrete’s top layer determines its resistance to scaling. Apart from maintenance, air-entraining and a correspondingly low water–cement (w/c) ratio are the key indicators. Air entraining (AE) improves the workability of a concrete mix, and also reduces sedimentation by making air bubbles retain the form of a suspended solid. Thus, AE increases frost resistance in two stages: it reduces bleeding; and, after scaling occurs, the top layer protects the concrete against the volumetric effects of freeze–thaw cycles. Reducing bleeding is very important because it can decrease the w/c value and, as a consequence, increase the strength of the top layer, resulting in protection against surface scaling [1,6,7,9,12,13,14,15].

The most important parameter of concrete mixes is the w/c ratio, which is responsible for their material properties and determines their durability and strength. In particular, lowering the w/c value reduces bleeding, increases strength, and consequently increases resistance to scaling. To ensure that concretes are resistant to scaling, concretes with a w/c ratio ≤ 0.30 should not have AE [1,2,6], while properly air-entrained concretes should have a w/c ratio that does not exceed 0.5 [1,4,6,9].

The AE meter is the proper air-entrained structure for hardened concrete, and protects against freeze–thaw cycles if ice forms in an air pore and is compressed by the surrounding matrix [7,16]. The basic parameter that characterizes the AE meter is the spacing factor (L) [8,17,18], which is the average distance at which each point in the paste will be protected against the formation of destructive scratches that can result from harmful expansion during internal crystallization [7,19]. Powers and Helmuth established that the critical L value lies in the range of 250–300 µm [6,7,8]. Modern guidelines limit this value to 200 µm [20,21].

According to EN 480-11 [22], the parameters that describe an air-entrained structure, i.e., the spacing factor (L), micro air content (A_300_), total content of air in hardened concretes (A), and specific surface area (α), take into account all of the pores in the concrete in the range of 10–4000 µm. This not only includes pores from air entraining, but also pores caught accidentally and pores that are a side effect of the use of superplasticizers (SPs) [23,24]. The third generation of SPs, which are based on modified polycarboxylates (MP) and reduce surface tension, cause air bubbles to form in the concrete mix and in concrete [25,26,27]. SPs based on MPs are formed by attaching side chains to the main chain of the polycarboxylate, which has strong steric activity. Due to the structure of the polymer chains, which contain special substituents, they simultaneously use two basic fluidization mechanisms: steric and electrostatic fluidization [28].

The results of tests on the impact of SPs on the air void parameters (AVPs) of hardened concrete showed [24] that liquefaction of a concrete mix by means of an SP reduces the effectiveness of the air-entraining admixture (AEA) due to an increase in the liquidity of the concrete mix, the action of the SP, interactions between the AEA and the SP, or the overlapping of these effects. In addition, an increase in the SP content, both in non-air-entrained concretes (NAECs) and air-entrained concretes (AECs), causes a decrease in the content of pores that is favorable for resistance to freeze–thaw cycles (entrained air) as against the content of unfavorable pores (entrapped air).

Khayat [29] showed that liquid mixtures, at w/c ratio values of 0.32, 0.40, and 0.45, may show a lack of resistance to scaling, and, in addition, the SP admixture may disrupt the air-entrained structure and cause an increase in the L value. On the other hand [30], the authors in showed that SP-liquefied concretes, when properly air-entrained, show resistance to scaling. The results of the research by Łaźniewska-Piekarczyk [25] showed that the air content in a self-compacting concrete (SCC) mix is a side effect of the SP and is very unstable. As a consequence, it shapes the AVPs, whose normally adopted values in concretes for the assessment of resistance to freeze–thaw cycles (L ≤ 0.200 mm and A_300_ ≥ 1.5% [1,21]) are not correlated with the tested resistance to freeze–thaw cycles of SCC concretes.

The impact of the side effect of SP use in the form of additional pores, as a consequence of shaping the air void structure (AVS) and the relationship with the concrete’s resistance to freeze–thaw cycles, has been the subject of many studies. These studies have particularly been concerned with SCC concretes with a very complex system of compatible admixtures and rheological problems [13,14,16,25,29,31,32,33,34,35,36,37,38,39,40,41]. Despite the numerous studies in this area, it is not yet possible to clearly determine the effect of an SP on the AVS due to the complexity of the parameters, particularly the rheological ones, that shape the AE of SCC concretes. Thus, the aim of this study was to unequivocally determine the influence of an SP based on an MP on the resistance to scaling of ordinary concretes and to assess this influence in the light of the obtained AVPs. This problem is extremely important because the requirements for the AVS are used as criteria for assessing a concrete’s resistance to scaling, which is disturbed by an MP-based SP.

## 2. Materials and Methods

To unambiguously determine the effect of an SP based on an MP, which is used to regulate the consistency of the concrete mix, tests were carried out on NAEC and AEC mixes and hardened concretes with the same basic recipe that differed only in the content of SP. The dose of AEA was held constant in the air-entrained mixtures.

We adopted commonly used SPs and AEAs in the research program to increase builders’ and engineers’ awareness and knowledge of the impact of SPs on a concrete’s resistance to scaling and its assessment in the light of AVPs. This research is particularly relevant to regions with a cold climate, as the concretes used in these regions must have adequate resistance to freeze–thaw cycles. Since an SP affects both the AVS in hardened AEC and NAEC and their resistance to scaling, it is important to determine its effect on both a concrete’s features and the relationships between them.

### 2.1. Examined Materials

#### 2.1.1. Cement and Aggregates

**Figure 1:**
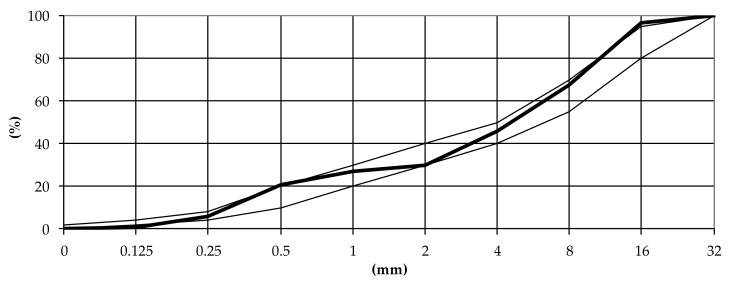
The grain size distribution of the aggregate.

Portland cement CEM I 42.5R with a density of 3.1 g/cm^3^ and a Blaine fineness of 3849 cm^2^/g, which meet the requirements for reference concrete in accordance with EN 480-1 [42], was used for the tests. The physical and chemical properties of the cement are given in Table 1. In the concrete mixes, a basalt aggregate with a maximum grain size of 16 mm and natural sand was used. The sand point was 29.6%. The grain size curve that meets the requirements for reference concrete in accordance with EN 480-1 for fine and coarse aggregate mixtures is given in Figure 1. Water was used in accordance with EN 1008.

#### 2.1.2. Chemical Admixtures

The properties of the admixtures are given in Table 2. The SP was a concentrated aqueous MP solution, while the AEA was a modified wood resin. The chemical compositions of the SP and the AEA are protected by the manufacturer’s patent.

The percentage content of SP and AEA was calculated in relation to the mass of the cement. The amount of SP was determined experimentally to obtain the consistency classes S1–S4. The amount of AEA in the air-entrained mixtures was held constant. All tests were conducted at laboratory temperature.

### 2.2. Proportions in and Preparation of Concrete Mixes

Eight NAEC and AEC mixes were made. The basic recipes, i.e., the content of cement, water, and coarse and fine aggregate, were the same (Table 3). In the NAEC mixes (Table 3), the SP content was variable and determined in order to obtain the required consistency classes (S1–S4). In the AEC mixes, the AEA content was held constant and was determined for a mix (C1) without SP, in which the air content was 6%.

The mixtures were made in a horizontal plan mixer with a volume of 0.1 m^3^. Coarse and fine aggregate and cement were mixed for 0.5 min, then some mixing water was added and the mixture was mixed for another 0.5 min. SP was added along with 2 dm^3^ of water and the mixture was stirred for 1 min. Finally, in the AEC mixtures, an AEA with 2 dm^3^ of water was added and the mixtures were mixed for 2 min. The total mixing time was 2 min for NAEC mixtures, and 4 min for AEC mixtures. Test samples were demolded after 24 h and stored in a chamber at 20 ± 2 °C and a humidity of 95 ± 5% in accordance with EN 12390-3 [43].

### 2.3. Tests on Fresh Concretes

At the concrete mix stage, consistency was tested by the Slump test method in accordance with EN 12350-2 [44], density was tested according to EN 12350-6 [45], and air content was tested by the pressure method according to EN 12350-7 [46]. The tests were carried out within 10 min of the ingredients being mixed together.

### 2.4. Scaling Test

A scaling resistance test was carried out in an automatic chamber for the freezing and thawing of samples according to CEN/TS 12390-9:2016 [47]. The test was conducted on eight series of four samples with dimensions of 140 × 72 × 50 mm, which were subjected to 56 freeze–thaw cycles in the presence of 3% NaCl. After 7, 14, 21, 28, 35, 42, 49, and 56 cycles, mass loss was measured.

To assess the resistance of concrete to scaling, the criteria from the Swedish standard of the Borås method SS 137,244 [6] were used. The standard categories of scaling resistance in the presence of 3% NaCl are:

- very good quality concrete: m_56_ < 0.1 kg/m^2^;
- good quality concrete: m_56_ < 0.2 kg/m^2^ or m_56_ < 0.5 kg/m^2^ and m_56_/m_28_ < 2 or m_112_ < 0.5 kg/m^2^;
- acceptable quality concrete: m_56_ < 1.0 kg/m^2^ and m_56_/m_28_ < 2 or m_112_ < 1.0 kg/m^2^; and
- unacceptable quality concrete: m_56_ > 1.0 kg/m^2^ and m_56_/m_28_ > 2 or m_112_ > 1.0 kg/m^2^.

### 2.5. Compressive Strength and Density

Compressive strength and density were determined after 28 days in accordance with EN 12390-3 [43] and EN 12390-7 [48], respectively. For each series of concretes, the tests were carried out on three 150 × 150 × 150 mm samples.

### 2.6. Air Void Characteristics

**Figure 2:**
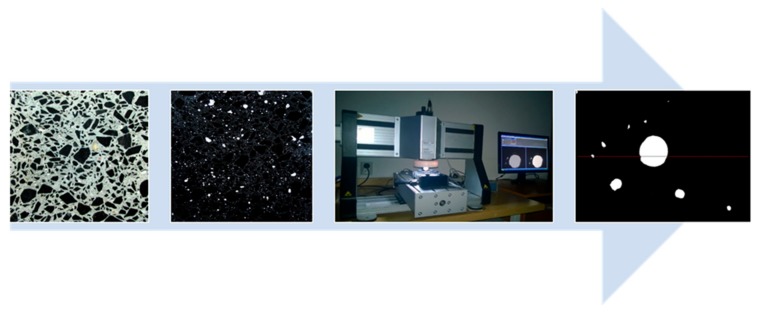
The air void parameter (AVP) determination scheme.

The AVPs were determined in accordance with EN 480-11 [22] by means of a RapidAir 457 automatic image analysis system for voids in hardened concrete (Figure 2). For each series of concretes, the test was carried out on two 150 × 150 × 20 mm samples. As a result of the analysis, the following air void parameters were obtained: total content of air in hardened concrete, A; specific surface area, α; spacing factor, L; and micro air content, A_300_.

## 3. Results

### 3.1. The Results of the Concrete Mix Tests.

The results of the concrete mix tests are given in Table 4. The consistency classes of both the NEAC and the AEC mixes varied from S1 to S4 in accordance with EN 206 [49]. The densities of the NAEC mixes ranged from 2567 to 2613 kg/m^3^; the densities of the AEC mixes ranged from 2470 to 2570 kg/m^3^. In the NAEC mixtures, the air content ranged from 1.2 to 2.9%; in the AEC mixtures, the air content ranged from 2.7 to 6.0%.

Liquefaction of concrete mixes with the SP resulted in an increase in density and a decrease in air content in both the AEC mixes and the NAEC mixes.

### 3.2. Compressive Strength and Density

The results of the compressive strength and density tests are given in Table 5.

### 3.3. Salt Scaling

The results of the scaling resistance tests are given in Table 6 and Table 7. All of the AEC mixes showed no scaling and, hence, were classified as very good quality concretes. In the NAEC mixes, the scaling mass was found to increase as the SP content increased. The NEAC without SP (C1) was found to be of very good quality, C2 and C3 were found to be of acceptable quality, and C4 was found to be of unacceptable quality.

### 3.4. The Air Void Parameters

The AVPs A, L, α, and A_300_ for the tested concretes are given in Table 8.

## 4. Discussion

### 4.1. The Effect of the SP on the Scaling Resistance

**Figure 3:**
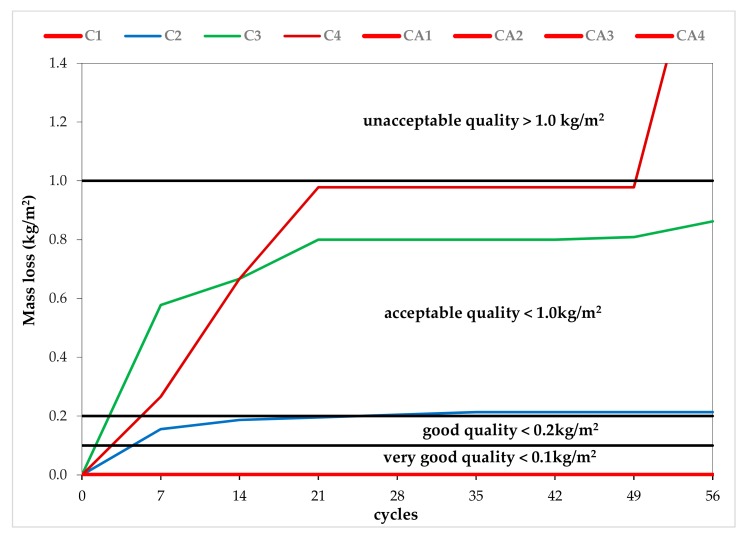
Mass loss in the concretes after 7, 14, 21, 28, 35, 42, 49, and 56 cycles.

**Figure 4:**
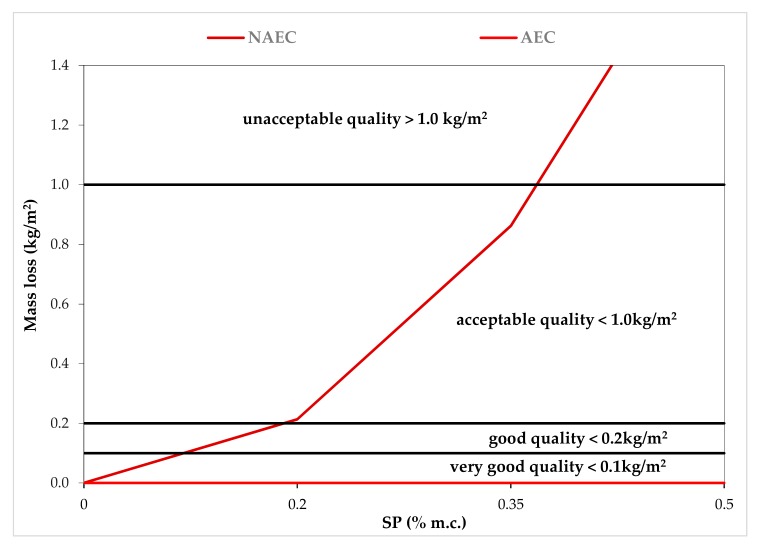
Dependence of mass loss after 56 cycles of scaling on superplasticizer (SP) content in the non-air-entrained concrete (NAEC) and air-entrained concrete (AEC) mixes.

In the AEC mixes, it was not possible to observe the effect of the SP on scaling resistance, because all of the AEC mixes, after 56 cycles of freezing and thawing in the presence of 3% NaCl, showed very good scaling resistance. However, in the NAEC mixes, a decrease in scaling resistance was observed as the SP content increased (Figure 3 and Figure 4). The NAEC mix without SP (the C1 series), similar to the AEC mixes, showed very good resistance to scaling. In C2, C3, and C4, we observed increases in the mass of flakes during the first seven test cycles (Figure 2). The most intense destruction occurred during the period from the 7th to the 21st cycle in all three concretes. In C2 and C3, after 21 cycles, we did not observe significant increases in the mass of scaling. C4 (with the highest dose of SP) was completely destroyed during the period between the 49th and 56th cycles (the sample disintegrated).

The destruction of C2 and C3 was in the form of a spatter on the concrete’s surface and, according to the literature [1,6,8,9,10], covered only the surface layer of the hardened cement paste, which has a thickness of several millimeters. However, in C4, scaling of the top layer’s surface took place until the 49th cycle and was followed by volumetric destruction.

As shown in Figure 4, with the increase in SP content and simultaneous liquefaction of the NAEC, the resistance to scaling decreased as a result of the surface layer of the concrete being weakened [1,6,7,9,12,13,14,15]. The surface layer of the concrete was strengthened by AE in the AEC mixes, ensuring that these concretes had freeze–thaw resistance during a very intensive 56-cycle scaling test. The resistance of the top layer, which is responsible for the resistance to scaling, was not found to be correlated with an increase in compressive strength (Table 5). As the SP content increased, the compressive strength of the concrete increased in both the NAEC and AEC mixes.

### 4.2. Assessment of Freeze–Thaw Resistance in the Light of Pore Structure Parameters

**Figure 5:**
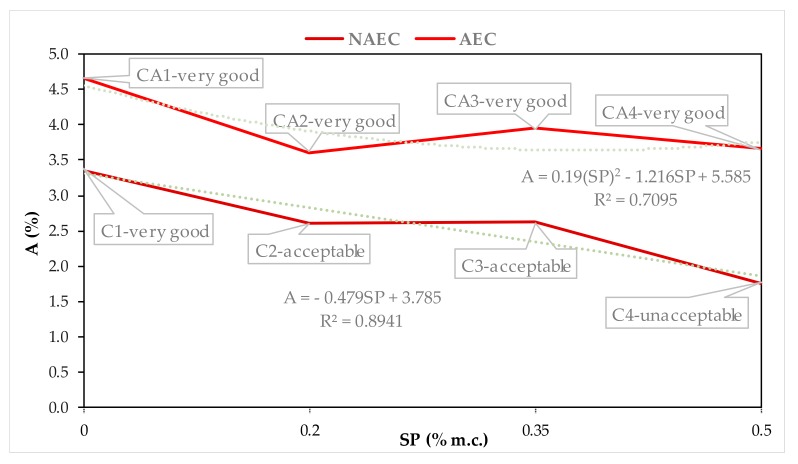
Dependences between A and SP contents in NAEC and AEC.

**Figure 6:**
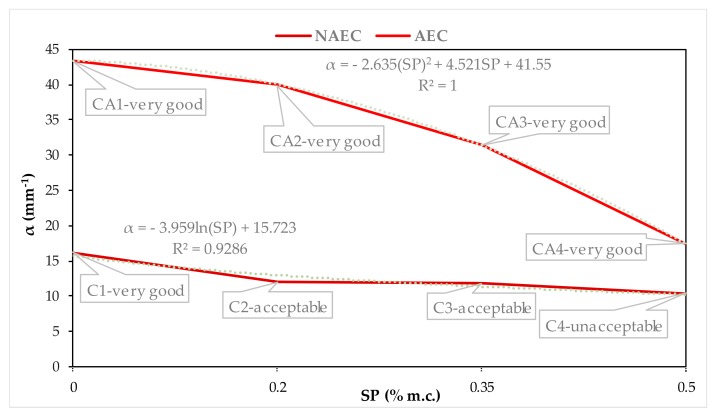
Dependences between α and SP contents in NAEC and AEC.

**Figure 7:**
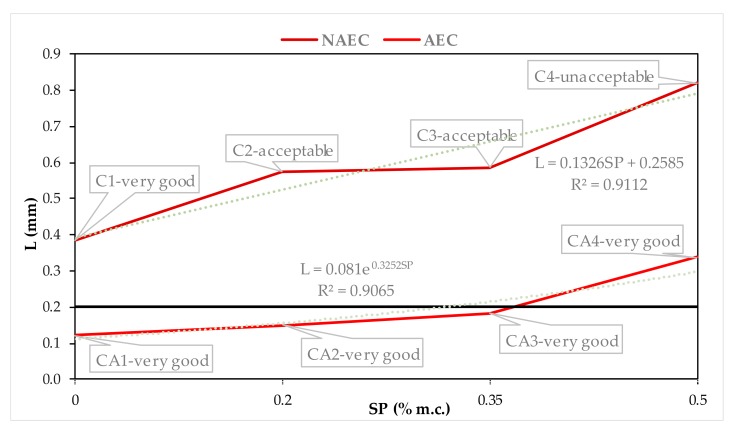
Dependences between L and SP contents in NAEC and AEC.

**Figure 8:**
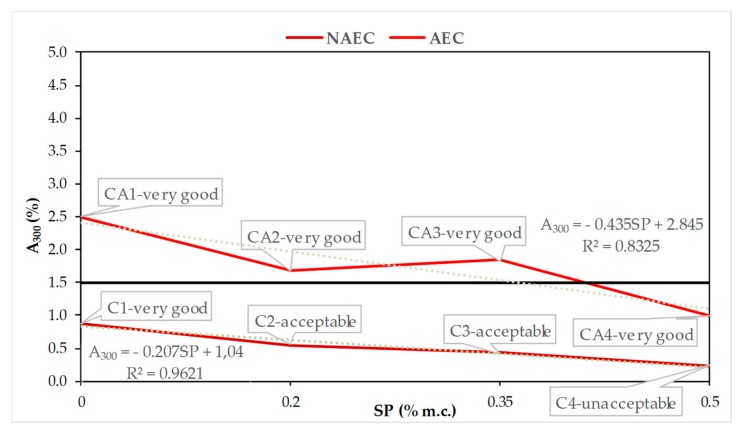
Dependences between A_300_ and SP contents in NAEC and AEC.

In order to determine the air void system in hardened concrete that will protect against freeze–thaw cycles [7,16], Figure 5, Figure 6, Figure 7 and Figure 8 show the quantitative, described by mathematical equations and quantitative relationships of AVP and SP in both the NEAC and AEC mixes together with an assessment of scaling [6].

The use of constant AE content in AEC mixtures CA1–CA4 (Table 8) resulted in a 1.0–1.9% increase in total air content in the hardened concretes as compared to NAEC mixtures C1–C4 (Figure 5). The highest increase was obtained in the concrete with the highest dose of SP. It should be noted that the use of SP, together with the liquefaction of the concrete mix, also caused a decrease in the air content in the NAEC mixes, while in the AEC mixes the air content remained at a comparable level. A ≥ 4% is recommended according to EN 206 to ensure concrete durability in exposure classes XF2–XF4. According to the American Concrete Institute (ACI) [4] for ensuring frost resistance air content in concrete depends on the maximum aggregate grain and fluctuate in the range of 4–7%. AEC without SP-CA1 meets the above requirements regarding the minimum air content. SP in CA2–CA4 concrete reduced the air content A to below the required minimum. However, the obtained very good resistance to scaling of these concretes (Figure 5) states that the total air content is not a key feature determining the resistance to scaling. Obtained test results show different results than those indicated by Łaźniewska [25] in SCC concretes, consisting in lowering and not increasing air content in concretes due to the SP action. This is probably described in [23,24] the rheological effect resulting from the liquefaction of the concrete mix.

Figure 6 shows the quantitative, described by mathematical equations and quantitative dependence between the specific surface area α and SP content. In the NAEC mixes, the specific surface area decreased slightly due to the use of SP and was comparable in all NAEC mixes with SP. Specific surface—α is a measure of the pore size. According to ACI recommendations [4,11], in concrete resistant to frost, its value should exceed 24 mm^−1^. The obtained α values in all NAECs, despite the variable resistance to scaling in the C1-very good to C4-unacceptable range, did not exceed 24 mm^−1^. Suggesting according to the guidelines [4,11] no resistance to scaling. In AEC, α significantly decreases (α = −2.635 (SP)^2 + 4.521SP + 41.55; R^2^ = 1) with increasing SP content. Which, with a slightly changing total air content A, confirms that SP causes an increase in the content of large pores. This is the effect of joining small pores into large ones due to a decrease in viscosity resulting from liquefaction of the concrete mix. In AEC CA1–CA3 the value of α exceeded the recommended 24 mm^−1^. Only in CA4 concrete the value of 17.45 mm^−1^ was obtained, with very good resistance to scaling. Therefore, the recommended level of α [4,11] has not been confirmed in the result disturbed by SP. A very significant impact of SP on the decrease in specific surface area was noted in the AEC mixes, which translates into an increase in the L parameter as SP increases in AEC (Figure 7).

Figure 7 shows the quantitative, described by mathematical equations, and quantitative relationship between the basic parameter that characterizes an air void system—L [8,17,18] and SP content. Its critical value at 200 µm is also marked in the figure [20,21]. In the NAEC mixes, the L values exceeded 0.200 mm. The AEC mixes CA1–CA3 had distribution ratios below 0.200 mm and were found to be very good quality concretes from a scaling assessment standpoint, thus confirming the literature data [1,21]. The AEC mix CA4, despite an L value of 0.338, which significantly exceeds the established criterion, showed very good resistance to scaling. SP caused the L parameter in both the AEC and NAEC mixes to increase, which indicates an increase in the spacing between pores. The parameter L confirms the tendency of small pores to join together into thick ones, signaled by parameter α and resulting in an increase in their spacing and expressed by an increase in the L parameter in both AEC and NAEC.

In Figure 8, we can observe a decrease in the number of pores with dimensions below 300 µm, which are most favorable for ensuring freeze–thaw resistance in both AEC and NAEC. The quantitative decreases in A_300_ as the SP content increases are described by linear relationships (Figure 8). Its critical value was determined to be 1.5% [20,21]. All NAEC mixes had an A_300_ value less than 1.5%. Similar to the L parameter, CA1–CA3 meet the required criterion and have an A_300_ value greater than 1.5%. The CA4 concretes, despite not meeting the A_300_ range requirement, showed very good freeze–thaw resistance.

The obtained AVP values confirm the literature data on the impact of SPs based on MPs on the decrease in pore content favorable for freeze–thaw resistance (entrained air) as against unfavorable pore content (entrapped air). They also confirm that AVPs take into account both air-entraining pores and pores that are a side effect of SP action [23,24]. As a consequence, SPs shape the AVPs, whose normally adopted values in concrete for the assessment of frost resistance (L ≤ 0.200 mm and A_300_ ≥ 1.5%) are not correlated with the tested freeze–thaw resistance in the case of ordinary concretes.

## 5. Conclusions

All AEC mixes, after 56 cycles of freezing and thawing in the presence of 3% NaCl, showed very good scaling resistance; therefore, no effect of SP on their scaling resistance was observed.

In the NAEC mixes, with an increase in SP content and simultaneous liquefaction, the resistance to scaling decreased as a result of the top layer of the concrete being weakened and changes in the porosity structure.

SP was found to significantly change the AVPs, particularly in the AEC mixes. It causes a significant decrease in the specific surface area, which results in an increase in the spacing factor L and a decrease in the content of micropores A_300_.

The qualitative analysis the impact of SPs based on MP on the AVPs: A, α, L, and A_300_ showed the decrease in pore content favorable for frost resistance (entrained air) as against unfavorable pore content (entrapped air). They also confirm that AVPs take into account both air-entraining pores and pores that are a side effect of SP action. As a consequence, SPs shape the AVPs, whose standard values in concretes for the assessment of freeze–thaw resistance (L ≤ 0.200 mm and A_300_ ≥ 1.5%) are not correlated with the tested scaling resistance in the case of ordinary concretes.

The quantitative, described by mathematical equations relationships of AVPs: A, α, L, and A_300_ and SP content in both NAEC and AEC were determined and associated with scaling resistance. These relationships can be used in more complex systems to be used in projects involving air-entrained SCC and pavement concretes.

## Figures and Tables

**Table 1:** The physical and chemical properties of CEM I 42.5R.

Setting Time, Vicat Test (min)		Water Demand (%)	Compressive Strength (MPa)	Chemical Analyses (%)			Loss on Ignition (%)
Initial	Final			**SO_3_**	**Cl**	**Na_2_O_eq_**	
184	242	27.1	58.2	2.90	0.091	0.64	3.58

**Table 2:** The properties of the admixtures.

Property	SP	AEA
Main base	Modified polycarboxylates	Modified wood resin
Specific gravity at 20 °C (g/cm^3^)	1.07 ± 0.02	1.02
pH value at 20 °C	4.4 ± 1	12.5
Chloride ion content (% mass)	<0.1	≤0.1
Alkali content (Na_2_O_eqiv._) (% mass)	≤0.6	<2

**Table 3:** The components of the concretes.

Name of Concrete	CEM I 42.5R (kg/m^3^)	w/c	Sand 0/2 mm (kg/m^3^)	Gravel 2/8 mm (kg/m^3^)	Gravel 8/16 mm (kg/m^3^)	Volume of Paste (%)	SP	AEA
							The Dosage of Admixtures by Weight of Cement (%)	
C1	391	0.46	572	795	635	30.71	–	–
CA1	391	0.46	572	795	635	30.71	–	0.20
C2	391	0.46	572	795	635	30.71	0.20	–
CA2	391	0.46	572	795	635	30.71	0.20	0.20
C3	391	0.46	572	795	635	30.71	0.35	–
CA3	391	0.46	572	795	635	30.71	0.35	0.20
C4	391	0.46	572	795	635	30.71	0.50	–
CA4	391	0.46	572	795	635	30.71	0.50	0.20

**Table 4:** The results of the concrete mix tests.

Name of Concrete	Slump (mm)	Density (kg/m^3^)	Vp (%)
C1	30	2567	2.9
CA1	10	2470	6.0
C2	70	2593	1.7
CA2	110	2515	4.6
C3	170	2613	1.2
CA3	180	2543	3.8
C4	210	2610	1.4
CA4	210	2570	2.7

**Table 5:** Results of the compressive strength and density tests.

Name of Concrete	f_c_ (MPa)	D (kg/m^3^)
C1	51.7	2532
CA1	49.3	2511
C2	58.6	2586
CA2	49.5	2498
C3	59.0	2556
CA3	52.5	2501
C4	59.0	2567
CA4	56.0	2525

**Table 6:** The decrease in mass after scaling with de-icing salt after different numbers of freeze–thaw cycles (kg/m^2^).

Name of Concrete	7 Cycles	14 Cycles	21 Cycles	28 Cycles	35 Cycles	42 Cycles	49 Cycles	56 Cycles
C1	0	0	0	0	0	0	0	0
CA1	0	0	0	0	0	0	0	0
C2	0.16	0.19	0.20	0.20	0.21	0.21	0.21	0.21
CA2	0	0	0	0	0	0	0	0
C3	0.58	0.67	0.80	0.80	0.80	0.80	0.81	0.86
CA3	0	0	0	0	0	0	0	0
C4	0.27	0.67	0.98	0.98	0.98	0.98	0.98	> 1.00 *
CA4	0	0	0	0	0	0	0	0

* One sample was disintegrated.

**Table 7:** The quality of concretes after 56 cycles of scaling with de-icing salt according to SS 137244 [6].

Name of Concrete	56 Cycles
C1	very good
CA1	very good
C2	acceptable
CA2	very good
C3	acceptable
CA3	very good
C4	unacceptable
CA4	very good

**Table 8:** The air void parameter values.

Name of Concrete	A (%)	L (mm)	α (mm^−1^)	A_300_ (%)
C1	3.36	0.382	16.09	0.88
CA1	4.66	0.122	43.46	2.50
C2	2.61	0.573	12.03	0.55
CA2	3.61	0.149	39.98	1.69
C3	2.62	0.585	11.89	0.43
CA3	3.95	0.181	31.47	1.84
C4	1.76	0.820	10.30	0.23
CA4	3.66	0.338	17.45	1.00

## References

[1] Pigeon M., Marchand J., Pleau R. (1996). Frost resistant concrete. Constr. Build. Mater..

[2] Pleau R., Pigeon M. (2014). Durability of Concrete in Cold Climates.

[3] Fagerlund G. (2018). Frost Destruction of Concrete—A Study of the Validity of Different Mechanisms. Nord. Concr. Res..

[4] (1977). Guide to Durable Concrete. J. Am. Concr. Inst..

[5] Fagerlund G. (1999). Service Life with Regard to Frost Attack—A Probabilistic Approach. Durability of Building Materials and Components 8.

[6] Valenza J.J., Scherer G.W. (2007). A review of salt scaling: I. Phenomenology. Cem. Concr. Res..

[7] Valenza J.J., Scherer G.W. (2007). A review of salt scaling: II. Mechanisms. Cem. Concr. Res..

[8] Powers T.C. (1969). The Properties of Fresh Concrete.

[9] Pigeon M., Talbot C., Marchand J., Hornain H. (1996). Surface microstructure and scaling resistance of concrete. Cem. Concr. Res..

[10] Tsang C., Shehata M.H., Lotfy A. (2016). Optimizing a Test Method to Evaluate Resistance of Pervious Concrete to Cycles of Freezing and Thawing in the Presence of Different Deicing Salts. Materials.

[11] Attiogbe E.K. (1996). Predicting freeze-thaw durability of concrete—A new approach. ACI Mater. J..

[12] Crumpton C.F., Jayaprakash G.P. (1982). Entrained air voids in concrete help prevent salt damage. Civ. Eng..

[13] Kang Y., Hansen W., Borgnakke C. (2012). Effect of air-void system on frost expansion of highway concrete exposed to deicer salt. Int. J. Pavement Eng..

[14] Mayercsik N.P., Vandamme M., Kurtis K.E. (2016). Assessing the efficiency of entrained air voids for freeze-thaw durability through modeling. Cem. Concr. Res..

[15] Sun Z., Scherer G.W. (2010). Effect of air voids on salt scaling and internal freezing. Cem. Concr. Res..

[16] Grubesa I.N., Markovic B., Vracevic M., Tunkiewicz M., Szenti I., Kukovecz A. (2019). Pore Structure as a Response to the Freeze/Thaw Resistance of Mortars. Materials.

[17] Attiogbe E.K. (1997). Volume fraction of protected paste and mean spacing of air voids. ACI Mater. J..

[18] Attiogbe E.K., Hoover K.C., Natesaiyer K., Simon M., Snyder K. (1994). Mean spacing of air voids in hardened concrete. ACI Mater. J..

[19] Coussy O., Monteiro P.J.M. (2008). Poroelastic model for concrete exposed to freezing temperatures. Cem. Concr. Res..

[20] Greene T.M. (2013). Chemical Admixtures for Concrete.

[21] Hasholt M.T. (2014). Air void structure and frost resistance: A challenge to Powers’ spacing factor. Mater. Struct..

[22] (2008). EN 480-11: Admixtures for concrete, mortar and grout-Test methods-Part 11: Determination of air void characteristics in hardened concrete.

[23] Plante P., Pigeon M., Saucier F. (1989). Air-void stability 2. influence of superplasticizers and cement. ACI Mater. J..

[24] Nowak-Michta A. (2015). Influence of superplasticizer on porosity structures in hardened concretes. Procedia Eng..

[25] Lazniewska-Piekarczyk B. (2012). The influence of selected new generation admixtures on the workability, air-voids parameters and frost-resistance of self compacting concrete. Constr. Build. Mater..

[26] Lazniewska-Piekarczyk B. (2014). The methodology for assessing the impact of new generation superplasticizers on air content in self-compacting concrete. Constr. Build. Mater..

[27] Wilinski D., Lukowski P., Rokicki G. (2016). Polymeric superplasticizers based on polycarboxylates for ready-mixed concrete: Current state of the art. Polimery.

[28] Łukowski P. (2016). Admixtures for concrete—Problem of compatibility. Materiały Budowlane.

[29] Khayat K.H. (1995). Frost durability of concrete containing viscosity-modifying admixtures. ACI Mater. J..

[30] Bordeleau D., Pigeon M., Banthia N. (1992). Comparative-study of latex-modified concretes and normal concretes subjected to freezing and thawing in the presence of a deicer salt solution. ACI Mater. J..

[31] Balaguru P., Ramakrishnan V. (1988). Chloride permeability and air void characteristics of concrete containing high range water reducing admixture. Cem. Concr. Res..

[32] Barfield M., Ghafoori N. (2012). Air-entrained self-consolidating concrete: A study of admixture sources. Constr. Build. Mater..

[33] Beaupre D., Lacombe P., Khayat K.H. (1999). Laboratory investigation of rheological properties and scaling resistance of air entrained self-consolidating concrete. Mater. Struct..

[34] Ding B., Liu J., Liu J., Sun W., van Breugel K., Miao C., Ye G., Chen H. (2008). Air bubble stability mechanism of air-entraining admixtures and air void analysis of hardened concrete. International Conference on Microstructure Related Durability of Cementitious Composites.

[35] Pigeon M., Langlois M. (1991). Study on frost-resistance of superplasticized concrete. Can. J. Civ. Eng..

[36] Zhang P., Liu G.G., Pang C.M., Yan X.L., Qin H.G. (2017). Influence of pore structures on the frost resistance of concrete. Mag. Concr. Res..

[37] Lazniewska-Piekarczyk B. (2013). The type of air-entraining and viscosity modifying admixtures and porosity and frost durability of high performance self-compacting concrete. Constr. Build. Mater..

[38] Szwabowski J., Lazniewska-Piekarczyk B. (2008). The importance of porosity structure parameters of freeze—thaw resistant self-compacting concrete. Cem. Wapno Beton.

[39] Lazniewska-Piekarczyk B. (2013). Examining the possibility to estimate the influence of admixtures on pore structure of self-compacting concrete using the air void analyzer. Constr. Build. Mater..

[40] Lazniewska-Piekarczyk B. (2013). The frost resistance versus air voids parameters of high performance self compacting concrete modified by non-air-entrained admixtures. Constr. Build. Mater..

[41] Lazniewska-Piekarczyk B. (2013). Effect of viscosity type modifying admixture on porosity, compressive strength and water penetration of high performance self-compacting concrete. Constr. Build. Mater..

[42] (2014). EN 480-1: Admixtures for concrete, mortar and grout–Test methods–Part 1: Reference concrete and reference mortar for testing.

[43] (2009). EN 12390-3: Testing hardened concrete–Part 3: Compressive strength of test specimens.

[44] (2009). EN 12350-2: Testing fresh concrete–Part 2: Slump-test.

[45] (2009). EN 12350-6: Testing fresh concrete–Part 6: Density.

[46] (2009). EN 12350-7: Testing fresh concrete–Part 7: Air content–Pressure methods.

[47] (2016). CEN/TS 12390-9:2016 Testing hardened concrete. De-icing salts were used to test the freeze–thaw resistance.

[48] (2009). EN 12390-7: Testing hardened concrete–Part 7: Density of hardened concrete.

[49] (2014). EN 206: Concrete-Specification, performance, production and conformity.

